# Severe hepatobiliary morbidity is associated with *Clonorchis sinensis* infection: The evidence from a cross-sectional community study

**DOI:** 10.1371/journal.pntd.0009116

**Published:** 2021-01-28

**Authors:** Men-Bao Qian, Hong-Mei Li, Zhi-Hua Jiang, Yi-Chao Yang, Ming-Fei Lu, Kang Wei, Si-Liang Wei, Yu Chen, Chang-Hai Zhou, Ying-Dan Chen, Xiao-Nong Zhou

**Affiliations:** 1 National Institute of Parasitic Diseases, Chinese Center for Disease Control and Prevention, Shanghai, China; 2 Chinese Center for Tropical Diseases Research, Shanghai, China; 3 Key Laboratory of Parasite and Vector Biology, Ministry of Health, Shanghai, China; 4 National Center for International Research on Tropical Diseases, Ministry of Science and Technology, Shanghai, China; 5 WHO Collaborating Center for Tropical Diseases, Shanghai, China; 6 School of Global Health, Chinese Center for Tropical Diseases Research-Shanghai Jiao Tong University School of Medicine, Shanghai, China; 7 Guangxi Center for Disease Control and Prevention, Nanning, China; 8 Hengxian People’s Hospital, Hengxian, China; 9 Hengxian Center for Disease Control and Prevention, Hengxian, China; Istituto Superiore di Sanità, ITALY

## Abstract

*Clonorchis sinensis* infection is highly prevalent in Asia. Diverse hepatobiliary morbidity has been documented for *C*. *sinensis* infection. This study aimed to assess the association between *C*. *sinensis* infection and hepatobiliary morbidity, taking into consideration of the control, confounders and infection intensity. A cross-sectional community survey was implemented in Hengxian county, southeastern China. Helminth infections were detected by fecal examination. Physical examination and abdominal ultrasonography were then conducted. After excluding confounding effects from gender, age and alcohol drinking, quantitative association between *C*. *sinensis* infection and hepatobiliary morbidity was assessed, and the effect from infection intensity was also evaluated, through adjusted odds ratio (aOR) and 95% confidence intervals (95% CI). 696 villagers older than 10 years were enrolled. The prevalence and infection intensity of *C*. *sinensis* were higher in male, elder people and the individuals consuming alcohol. Light *C*. *sinensis* infection was associated with the increase of diarrhoea (aOR: 2.2, 95% CI: 1.1–4.5). *C*. *sinensis* infection was associated with the increase of fatty liver (aOR: 2.7, 95% CI: 1.4–5.2), and the effect was similar in different infection intensities. Moderate *C*. *sinensis* infection was associated with the increase of gallbladder stone (aOR: 3.0, 95% CI: 1.1–8.6), while moderate and heavy infections with the increase of intrahepatic bile duct dilatation (aOR: 2.2, 95% CI: 1.0–4.9 and aOR: 4.3, 95% CI: 1.9–9.9, respectively). *C*. *sinensis* infection had an effect on the development of periductal fibrosis (aOR: 3.2, 95% CI: 2.1–4.9), which showed increasing trend by infection intensity. The length and width of gallbladder in those with *C*. *sinensis* infection were enlarged, especially in those over 30 years old. *C*. *sinensis* infection is significantly associated with hepatobiliary morbidity. The occurrence of some morbidity was strongly related to the infection intensity. Awareness on harm of clonorchiasis should be raised both for policy-makers and villagers to adopt effective interventions.

## Author summary

Clonorchiasis is caused by ingestion of raw freshwater fish, which carries the larvae of *Clonorchis sinensis*. In this study, we aimed to assess the damages associated with clonorchiasis. A cross-sectional field survey was carried out in Hengxian county, southeastern China. Stool samples were collected and examined for helminth infections. Then, physical examination and abdominal ultrasonography were conducted. In total, 696 villagers older than 10 years were included. The prevalence and infection intensity of *C*. *sinensis* were higher in male, elder people and those drinking alcohol. Excluding the impact from gender, age and alcohol drinking, it was demonstrated that light *C*. *sinensis* infection was associated with the increase of diarrhoea, moderate infection with gallbladder stone, and moderate and heavy infections with intrahepatic bile duct dilatation. *C*. *sinensis* infection was relevant to the increase of fatty liver, and the effect was similar in different infection intensities. *C*. *sinensis* infection was associated with the development of periductal fibrosis and the effect increased by infection intensity. The length and width of gallbladder in individuals with *C*. *sinensis* infection were enlarged, especially in those over 30 years old. Thus, our study demonstrates that *C*. *sinensis* infection is associated with severe hepatobiliary morbidity, and the effect is strongly related to infection intensity for some morbidity. Concerted efforts should be exerted to tackle the morbidity in clonorchiasis endemic areas.

## Introduction

Clonorchiasis is caused by the ingestion of raw freshwater fish, which contains the infective larvae of *Clonorchis sinensis* [1,2]. Clonorchiasis is one important food-borne trematodiasis [3]. An estimation of 15 million cases with clonorchiasis distribute in China, the Republic of Korea, northern Vietnam and parts of Russia, where habit of ingesting raw freshwater fish is deeply rooted [1,2,4]. Particularly, about 13 million cases are estimated in China [1,2].

Adult worms of *C*. *sinensis* parasitize in intrahepatic bile ducts [5,6]. In some cases with high number of worms, the adults may migrate to the gallbladder [5]. *C*. *sinensis* eggs are expelled with the faeces, and thus fecal examination for eggs is usually applied for definite diagnosis [2]. The number of eggs in faeces is directly related to the worm burden in hosts and thus the indicator of infection intensity by quantitating eggs is usually used to refer to the degree of infection [7].

*C*. *sinensis* infection predominantly leads to hepatobiliary abnormalities [8]. In early stage of infection, symptoms are usually mild, while some cases present diverse symptoms, especially diarrhoea and right upper quadrant pain [2]. Chronic *C*. *sinensis* infection could lead to various liver and biliary morbidity, among which cholelithiasis is frequently reported [9–12]. Particularly, *C*. *sinensis* is classified as definite carcinogen, causing fatal cholangiocarcinoma [13,14]. Although diverse morbidity has been reported to be associated with *C*. *sinensis* infection, most was documented in cases studies without control. Cofounding factors as gender and age are usually neglected. Especially, alcohol also causes liver conditions, and it is frequently consumed when people enjoy raw freshwater fish [15,16]. Furthermore, the impact of infection intensity on morbidity deserves to be explored. Thus, we carried out a cross-sectional community survey in southeastern China to assess the hepatobiliary morbidity associated with *C*. *sinensis* infection.

## Material and methods

### Ethics statement

The study was approved by the Ethics Committee of the National Institute of Parasitic Diseases, Chinese Center for Disease Control and Prevention in Shanghai, China (reference no. 2011–005). Written informed consent was obtained from all participants or their guardians for those aged <18 years. People infected with *C*. *sinensis* or soil-transmitted helminths were administrated free of charge with praziquantel (25 mg/kg, t.i.d, 2 days) and albendazole (400 mg, single oral dose), respectively.

### Study area and participants

This study was conducted between March and May 2012 in Hengxian county, Guangxi, China. Hengxian county is located in the southeast of China, where increasing prevalence of *C*. *sinensis* was documented [17]. Two villages named Xiaoling and Shangcha were included. Villagers over 5 years old were firstly invited to participate in fecal examination, and then those over 10 years old were again invited to join the ultrasound examination.

### Diagnosis of helminth infections

One stool sample was collected from each participant. Then both the Kato-Katz method and washing sedimentation technique were employed. Three Kato-Katz thick smears with a 41.7 mg template were prepared for each sample [18], in which *C*. *sinensis* eggs were recorded quantitatively and other helminth eggs qualitatively. One smear was examined for each sample in washing sedimentation technique [19], in which all helminth eggs were recorded qualitatively.

### Questionnaire and abdominal ultrasonography examination

The information on alcohol drinking, diarrhoea and right upper quadrant pain was obtained by a trained interviewer. Diarrhoea was defined as the occurrence of watery stool or over three times defecation per day during past one month. The timing of right upper quadrant pain also referred to the past one month. Three categories were provided for alcohol drinking, namely none, 1–7 times per week and over 7 times per week. Hepatomegaly and splenomegaly were detected through physical examination. Abdominal ultrasonographical examination (SSC-218, ALOKA, Tokyo, Japan) with a 3.5MHz probe was performed by an experienced radiologist. The size of gallbladder was measured, and the change of gallbladder wall (thickening or blur), sludge or polyps of gallbladder, cholecystectomy were recorded. Cholelithiasis was evaluated separately for intrahepatic bile duct, extrahepatic bile duct, and gallbladder. Duct dilation was assessed for intrahepatic bile duct (over 0.3 cm in internal diameter) and extrahepatic bile duct (over 0.8 cm in internal diameter) [20]. Periductal fibrosis was defined by periductal echoes (more than 3 mm) around second order intrahepatic bile duct [21]. Grading of the fibrosis included grade 0 = normal, grade I = echoes in 1 segment of the liver, grade II = 2–3 segments, and grade III = more than 3 segments [21]. Fatty liver, liver cyst and other abnormalities were also evaluated.

### Statistical analysis

Age was grouped into four categories, namely 10–29 years, 30–44 years, 45–59 years and 60+ years. Alcohol drinking was grouped into two categories, namely negative and positive (combination of those drinking 1–7 times per week and over 7 times per week), because those over 7 times per week were all infected with *C*. *sinensis* in this study. Helminth infection was classified as positive if one egg was detected in any of the three Kato-Katz thick smears and one washing sedimentation smear. In *C*. *sinensis*, eggs per gram of feces (EPG) was calculated by multiplying the average of eggs in three Kato-Katz thick smears by 24. Infection intensity of *C*. *sinensis* was then categorized as light (1–999 EPG), moderate (1000–9999 EPG), and heavy (≥10 000 EPG) [22]. If *C*. *sinensis* egg was detected in washing sedimentation technique other than the Kato-Katz method, then the intensity was classified as light infection, because EPG is usually very low in false negative individuals in the Kato-Katz method [23]. Periductal fibrosis was grouped into two categories, namely negative (grade 0) and positive (combination of grade I, II and III).

Multivariable logistic regression model was used to analyze the odds of infection, in which *C*. *sinensis* infection was set as outcome, and gender, age groups and alcohol drinking were all included as predictors. To analyze the odds of morbidity, univariable logistic regression model was first applied, in which each morbidity indicator was considered as outcome, and gender, age groups, alcohol drinking, *C*. *sinensis* infection (dichotomous variable including negative and positive categories) or *C*. *sinensis* intensity (polytomous variable including four categories, namely negative, light infection, moderate infection and heavy infection) as predictors, respectively. Then, one multivariable logistic regression model was used in which each morbidity indicator was set as outcome, and gender, age groups, alcohol drinking and *C*. *sinensis* infection simultaneously as predictors. Finally, another multivariable logistic regression model was employed in which single morbidity was set as outcome, and gender, age groups, alcohol drinking and *C*. *sinensis* intensity all as predictors. In univariable logistic regression, crude odds ratio (cOR) and 95% confidence intervals (95% CI) were provided, while adjusted odds ratio (aOR) and corresponding 95% CI were provided in multivariable logistic regression. General linear model was employed to compare the size of gallbladder, in which the length and width was set as dependent variable respectively while gender, age groups, alcohol drinking and *C*. *sinensis* infection as factors. If there existed interaction, further comparisons by *C*. *sinensis* infection stratified by gender, age groups and/or alcohol drinking were done.

## Results

### Helminth infections of participants

In total, 1295 villagers from two villages participated in fecal examination, out of which 696 accepted ultrasonography examination. Among these 696 villagers, 460 (66.1%), 5 (0.7%), 17 (2.4%), and 27 (3.9%) were infected with *C*. *sinensis*, *Ascaris lumbricoides*, *Trichuris trichiura*, and hookworm, respectively. In *C*. *sinensis*, 185 (40.2%), 158 (34.3%) and 117 (25.4%) belonged to light, moderate and heavy infections, respectively.

The epidemiological profiles of *C*. *sinensis* infection varied by gender, age groups and in those with different habits on alcohol drinking (**Table 1**). The aOR of *C*. *sinensis* infection was 6.0 (95% CI: 3.7–9.6) in male compared to female. Compared to those aged 10–29 years, the aOR was 4.6 (95% CI: 2.5–8.5), 6.4 (95% CI: 3.6–11.6) and 8.7 (95% CI: 4.7–16.0) in those aged 30–44 years, 45–59 years and 60+ years. The aOR was 3.4 (95% CI: 2.2–5.3) in individuals with alcohol drinking compared to those without.

**Table 1:** The prevalence of *Clonorchis sinensis* infection by gender, age groups and alcohol drinking.

Factors		No. participants	*C*. *sinensis* infection				*C*. *sinensis* infection intensity			
			No.	Percentage (%)	aOR (95% CI)^a^	P	Light (%)	Moderate (%)	Heavy (%)	P
**Gender**										
	**Female**	370	179	48.4	1.0		126 (70.4)	40 (22.3)	13 (7.3)	
	**Male**	326	281	86.2	6.0 (3.7–9.6)	<0.001	59 (21.0)	118 (42.0)	104 (37.0)	<0.001
**Age groups (years)**						<0.001				0.045
	**10–29**	113	47	41.6	1.0		24 (51.1)	19 (40.4)	4 (8.5)	
	**30–44**	167	113	67.7	4.6 (2.5–8.5)	<0.001	46 (40.7)	44 (38.9)	23 (20.4)	0.166
	**45–59**	224	155	69.2	6.4 (3.6–11.6)	<0.001	57 (36.8)	49 (31.6)	49 (31.6)	0.007
	**60+**	192	145	75.5	8.7 (4.7–16.0)	<0.001	58 (40.0)	46 (31.7)	41 (28.3)	0.021
**Alcohol drinking**^**b**^										
	**No**	364	178	48.9	1.0		108 (60.7)	42 (23.6)	28 (15.7)	
	**Yes**	330	280	84.8	3.4 (2.2–5.3)	<0.001	75 (26.8)	116 (41.4)	89 (31.8)	<0.001
**Total**		696	460	66.1	-	-	185 (40.2%)	158 (34.3%)	117 (25.4%)	-

a Gender, age groups and alcohol drinking were all included in multivariable logistic regression model.

b Data were not provided in two persons.

### Self-report morbidity and physical examination

The prevalence of diarrhoea was 15.4% and 5.9% in those with and without *C*. *sinensis* infection (cOR: 2.9, 95% CI:1.6–5.3) (**Tables 2** and **S1**). Overall, the aOR was 2.0 (95% CI: 1.0–4.0) in those with the infection compared to those without (**Table 3**). The aOR was also significant in those with light infection (2.2, 95% CI: 1.1–4.5) compared to non-infected individuals, but there existed no significant differences in moderate and heavy infections compared to those without *C*. *sinensis*.

**Table 2:** Morbidity in participants stratified by *Clonorchis sinensis* infection.

*C*. *sinensis* infection		No. with morbidity (%)															
Group	No.	Diarrhoea	Right upper quadrant pain	Hepatomegaly	Fatty liver	Liver cyst	Cholelithiasis	Gallbladder stone	Intrahepatic bile duct stone	Any bile duct dilatation	Intrahepatic bile duct dilatation	Extrahepatic bile duct dilatation	Periductal fibrosis^a^	Cholecystectomy	Polyps of gallbladder	Sludge of gallbladder	Changes in gallbladder wall
**Positive**	460	71 (15.4)	42 (9.1)	3 (0.7)	69 (15.0)	11 (2.4)	45 (9.8)	39 (8.5)	7 (1.5)	96 (20.9)	95 (20.7)	5 (1.1)	297 (65.1)	1 (0.2)	1 (0.2)	19 (4.1)	84 (18.3)
**Negative**	236	14 (5.9)	15 (6.4)	0 (0.0)	14 (5.9)	1 (0.4)	8 (3.4)	7 (3.0)	1 (0.4)	13 (5.5)	13 (5.5)	0 (0.0)	50 (21.2)	0 (0.0)	0 (0.0)	4 (1.7)	27 (11.4)
**Total**	696	85 (12.2)	57 (8.2)	3 (0.4)	83 (11.9)	12 (1.7)	53 (7.6)	46 (6.6)	8 (1.1)	109 (15.7)	108 (15.5)	5 (0.7)	347 (50.1)	1 (0.1)	1 (0.1)	23 (3.3)	111 (15.9)

a The total number of participants was 692, including 456 with *C*. *sinensis* infection and 236 without the infection.

**Table 3:** Association of morbidity and infection with *Clonorchis sinensis* adjusted for gender, age groups and alcohol drinking.

Morbidity	All infection		Light infection		Moderate infection		Heavy infection	
	aOR (95% CI)	P	aOR (95% CI)	P	aOR (95% CI)	P	aOR (95% CI)	P
**Diarrhoea**	2.0 (1.0–4.0)	0.039	2.2 (1.1–4.5)	0.033	1.7 (0.8–3.9)	0.184	2.2 (0.9–5.2)	0.082
**Fatty liver**	2.7 (1.4–5.2)	0.003	2.8 (1.4–5.5)	0.004	2.5 (1.1–5.7)	0.023	2.5 (1.0–6.3)	0.042
**Cholelithiasis**	2.4 (1.0–5.7)	0.044	2.3 (0.9–5.6)	0.079	2.8 (1.0–7.5)	0.045	2.4 (0.8–7.2)	0.113
**Gallbladder stone**	2.2 (0.9–5.6)	0.083	1.9 (0.7–5.2)	0.188	3.0 (1.1–8.6)	0.040	2.2 (0.7–7.1)	0.181
**Any bile duct dilatation**	1.9 (0.9–3.7)	0.072	1.2 (0.5–2.7)	0.638	2.2 (1.0–4.9)	0.047	4.4 (1.9–10.1)	<0.001
**Intrahepatic bile duct dilatation**	1.9 (0.9–3.7)	0.073	1.2 (0.6–2.7)	0.632	2.2 (1.0–4.9)	0.044	4.3 (1.9–9.9)	<0.001
**Periductal fibrosis**	3.2 (2.1–4.9)	<0.001	2.1 (1.3–3.3)	0.002	4.9 (2.9–8.4)	<0.001	13.3 (6.2–28.7)	<0.001
**Changes in gallbladder wall**	1.0 (0.6–1.8)	0.881	1.3 (0.8–2.4)	0.317	0.6 (0.3–1.2)	0.164	0.9 (0.4–1.9)	0.749

Although the prevalence of right upper quadrant pain and hepatomegaly in individuals with *C*. *sinensis* infection was slightly higher than those without infection, the differences were nonsignificant in both univariable and multivariable logistic regression analysis (**S2 Table**). Splenomegaly was not detected in any participant.

### Hepatobiliary morbidity

Fatty liver occurred in higher frequency in those with *C*. *sinensis* infection (15.0%) than those without (5.9%) (cOR: 2.8, 95% CI: 1.5–5.1) (**Tables 2** and **S3**). Compared to those without the infection, the overall aOR was 2.7 (95% CI: 1.4–5.2) in infected persons, which was also significant in all subgroups, namely 2.8 (95% CI: 1.4–5.5), 2.5 (95% CI: 1.1–5.7), 2.5 (95% CI: 1.0–6.3) in light, moderate and heavy infections, respectively (**Table 3**).

In total, cholelithiasis was found in 53 persons, with 46 in gallbladder and eight in intrahepatic bile duct (with one concurrent case in gallbladder) (**Table 2**). Overall, the aOR of cholelithiasis was 2.4 (95% CI: 1.0–5.7), which was 2.3 (95% CI: 0.9–5.6), 2.8 (95% CI: 1.0–7.5), 2.4 (95% CI: 0.8–7.2) in light, moderate and heavy infections, respectively, compared to non-infected individuals (**Tables 3** and **S4**). Compared to those without *C*. *sinensis*, the overall aOR of gallbladder stone was 2.2 (95% CI: 0.9–5.6), which was 1.9 (95% CI: 0.7–5.2), 3.0 (95% CI: 1.1–8.6), 2.2 (95% CI: 0.7–7.1) in light, moderate and heavy infections, respectively (**Tables 3** and **S5**).

Any bile duct dilatation was detected in 109 participants, including 108 intrahepatic and five extrahepatic bile duct dilatation (with four concurrent cases). The proportion was higher in those with *C*. *sinensis* infection (20.9%) compared to those without (5.5%) (cOR: 4.5, 95% CI: 2.5–8.3) (**S6 Table**). The overall aOR of any bile duct dilatation was 1.9 (95% CI: 0.9–3.7) in infected individuals compared to non-infected participants (**Table 3**). The aOR was not significant in light infection (1.2, 95% CI: 0.5–2.7), but significant in moderate infection (2.2, 95% CI: 1.0–4.9) and heavy infection (4.4, 95% CI: 1.9–10.1) compared to non-infected participants. The association of *C*. *sinensis* infection with intrahepatic duct dilatation was similar to that in any bile duct dilatation (**Tables 3** and **S7**).

Out of 692 participants with available data, 345 participants (49.9%) showed no periductal fibrosis, while those with periductal fibrosis by grade I, grade II, and grade III were 91 (13.2%), 141 (20.4%) and 115 (16.6%), respectively. Higher prevalence of periductal fibrosis was demonstrated in those with *C*. *sinensis* infection (65.1%) than those without (21.2%) (cOR: 6.9, 95% CI: 4.8–10.0) (**S8 Table**). The overall aOR of periductal fibrosis was 3.2 (95% CI: 2.1–4.9) in those infected participants, which was 2.1 (95% CI: 1.3–3.3), 4.9 (95% CI: 2.9–8.4) and 13.3 (95% CI: 6.2–28.7) in light, moderate and heavy infections, respectively, compared to non-infected persons (**Table 3**).

Although the changes in gallbladder wall was significantly higher in those with *C*. *sinensis* infection compared to those without infection in univariate logistic regression analysis (cOR: 1.7, 95% CI: 1.1–2.8), the difference was not significant in multivariate logistic regression model (aOR: 1.0, 95% CI: 0.6–1.8) (**Tables 3** and **S9**).

Although such morbidity indicators including liver cyst, intrahepatic bile duct stone, extrahepatic bile duct dilatation, cholecystectomy, polyps of gallbladder and sludge of gallbladder was detected, they showed no association with *C*. *sinensis* infection in both univariable and multivariable logistic regression analysis (**Tables 2** and **S2)**.

### Size of gallbladder

In female, the length and width of gallbladder in individuals without *C*. *sinensis* infection were 4.6 cm and 2.0 cm in aged 10–29 years, 4.9 cm and 2.2 cm in aged 30–44 years, 4.8 cm and 2.0 cm in aged 45–59 years, and 4.7 cm and 2.1 cm in aged 60+ years **(Table 4)**. The corresponding figures in infected individuals were 4.9 cm and 2.3 cm, 5.3 cm and 2.4 cm, 5.7 cm (P = 0.003) and 2.5 cm (P = 0.002), 5.9 cm (P = 0.001) and 2.7 cm (P = 0.002).

**Table 4:** Size of gallbladder in different groups^a^^b^.

Age groups	Female						Male					
	Negative			Positive			Negative			Positive		
	No.	Length (SD) (cm)	Width (SD) (cm)	No.	Length (SD) (cm)	Width (SD) (cm)	No.	Length (SD) (cm)	Width (SD) (cm)	No.	Length (SD) (cm)	Width (SD) (cm)
**10–29**	37	4.6 (0.9)	2.0 (0.5)	12	4.9 (1.6)	2.3 (0.7)	28	4.9 (1.1)	2.0 (0.4)	35	5.3 (1.4)	2.2 (0.7)
**30–44**	47	4.9 (1.0)	2.2 (0.5)	41	5.3 (1.5)	2.4 (0.8)	7	5.1 (1.2)	2.2 (0.8)	72^c^	7.0 (2.3)^d^	3.2 (1.2)^e^
**45–59**	65	4.8 (1.3)	2.0 (0.5)	62	5.7 (1.9)^f^	2.5 (0.8)^g^	3	6.5 (0.9)	2.0 (0.4)	90	7.5 (2.4)	3.4 (1.3)^h^
**60+**	40	4.7 (1.0)	2.1 (0.5)	64	5.9 (1.7)^i^	2.7 (0.9)^j^	7	5.3 (0.5)	2.5 (0.6)	81	6.4 (2.1)	3.1 (1.1)

a In the length, general linear model was significant (F = 13.9, P<0.001). In addition to main effects from gender (F = 17.2, P<0.001), age groups (F = 4.9, P = 0.002) and *C*. *sinensis* infection (F = 17.2, P<0.001), secondary order interaction (gender * age groups * *C*. *sinensis* infection) was also demonstrated (F = 2.8, P = 0.002). In the width, general linear model was significant (F = 15.2, P<0.001). In addition to main effects from gender (F = 7.2, P = 0.007), age groups (F = 5.1, P = 0.002) and *C*. *sinensis* infection (F = 30.1, P<0.001), secondary order interaction (gender * age groups * *C*. *sinensis* infection) was also demonstrated (F = 3.5, P<0.001).

b Data on length were not provided in five cases and data on width were not provided in six cases.

c The figure was 71 in width.

d Positive *vs* negative: F = 7.0, P = 0.008.

e Positive *vs* negative: F = 7.4, P = 0.007.

f Positive *vs* negative: F = 8.7, P = 0.003.

g Positive *vs* negative: F = 10.0, P = 0.002.

h Positive *vs* negative: F = 7.5, P = 0.006.

i Positive *vs* negative: F = 10.2, P = 0.001.

j Positive *vs* negative: F = 9.9, P = 0.002.

In male, the length and width of gallbladder in those non-infected persons were 4.9 cm and 2.0 cm in aged 10–29 years, 5.1 cm and 2.2 cm in aged 30–44 years, 6.5 cm and 2.0 cm in aged 45–59 years, and 5.3 cm and 2.5 cm in aged 60+ years **(Table 4)**. The corresponding figures in individuals with *C*. *sinensis* infection were 5.3 cm and 2.2 cm, 7.0 cm (P = 0.008) and 3.2 cm (P = 0.007), 7.5 cm and 3.4 cm (P = 0.006), 6.4 cm and 3.1 cm.

## Discussion

Compared to the low endemicity of soil-transmitted helminthiases, clonorchiasis was highly endemic in the two study villages, which was consistent to the county-level epidemiological survey [17]. The epidemiological profiles were characterized by a higher prevalence and infection intensity in male and elder people, which was same to other surveys [17,24–28]. Differences in the ingestion of raw freshwater fish determine the characteristics. There existed a strong relationship between *C*. *sinensis* infection and alcohol drinking. Several potential reasons might be responsible. Firstly, alcohol could help remove the fishy smell of raw fish. Secondly, ingestion of raw freshwater fish occurs frequently when people get together, and on this occasion alcohol is usually consumed. Thirdly, there exists a misunderstanding in some persons that alcohol could help kill the larvae of *C*. *sinensis* in raw fish [15]. Thus, it is necessary to exclude potential confounding effects from gender, age and alcohol drinking, to accurately assess the morbidity associated with *C*. *sinensis* infection, which was also justified in this study.

This study firstly establishes the relationship between *C*. *sinensis* infection and fatty liver. High proportion (11.9%) of participants developed fatty liver, and it was higher in those with *C*. *sinensis* infection (15.0%) compared to non-infected individuals (5.9%). Even the effects from other confounders (gender, age and alcohol drinking) were eliminated, the association was still significant in any infection intensity. High occurrence of fatty liver had also been documented in persons infected with *Opisthorchis viverrini*, e.g. 10.6% in Thailand [29] and 12.1% in Laos [30]. However, no control was included in those studies. In the genome of *C*. *sinensis*, the pathway for fatty acid metabolism is complete, while key genes involved in fatty acid biosynthesis are missing [31,32]. Thus, the worms need to receive lipids from the bile of the hosts. *C*. *sinensis* fatty acid binding protein is highly expressed in the worms, and it plays an important role in the intracellular transport of long-chain fatty acids obtained from the hosts [33,34]. In mouse model, many genes involved in fatty acid metabolism are downregulated in the livers of mouse infected with *C*. *sinensis*, which is argued to inhibit the use of fatty acids by hosts and thus increase the use by the worms [35]. It has also been observed that fatty drops appear in the liver of guinea-pigs after infection with *C*. *sinensis* [36]. These evidences support our finding that *C*. *sinensis* infection is associated with the development of fatty liver. However, more researches are needed to further verify the etiological association and illuminate the mechanism.

Cholelithiasis is frequently presented in individuals infected with *C*. *sinensis* [9–12]. In one case-control study, the occurrence of intrahepatic bile duct stone increased in those with *C*. *sinensis* infection, which was diagnosed based on radiological evidence other than fecal examination [9]. However, in the same study, the occurrence of extrahepatic bile duct stone and gallbladder stone was not associated with *C*. *sinensis* infection. In another case study, *C*. *sinensis* infection was argued to cause gallbladder stone, because *C*. *sinensis* infection weakens the function of gallbladder, and causes the precipitation of bilirubinate, calcium carbonate crystals, and mucin on *C*. *sinensis* eggs [10]. Our study adds further evidence on the association between *C*. *sinensis* infection and cholelithiasis.

Bile duct dilatation is important imaging appearance in clonorchiasis [37,38]. Adult worms of *C*. *sinensis* usually parasitize in intrahepatic bile duct [5,6]. Thus, it is reasonable that high occurrence of dilatation was recorded in intrahepatic other than extrahepatic bile duct. High infection intensities indicate more worm burden in bile duct, and thus the increase of dilatation in moderate and heavy infections is sound. On comparison, dilatation has not yet developed in individuals with light infection.

The development of periductal fibrosis is complex. The mechanical obstruction and injury of bile duct by the worms, toxic effects of the worms’ excretory-secretory products, and subsequent immunopathology by inflammation comprehensively contribute to the development of periductal fibrosis in hosts infected by *C*. *sinensis* [2]. Thus, the worm burden is directly related to the damage, which explains the increasing trend of periductal fibrosis by infection intensity. Periductal fibrosis is early indicator in the development of cholangiocarcinoma [39]. However, this study didn’t detect liver mass lesions suspected for cholangiocarcinoma. That is probably attributable to the relative low incidence of cholangiocarcinoma [1] and limited sample size in this study.

Diarrhoea is a frequently reported symptom in those individuals with clonorchiasis [2,40–42]. In this study, the relationship between *C*. *sinensis* infection and diarrhoea was also established. However, the effect was only demonstrated significantly in light infection. Although right upper quadrant pain is also another frequent symptom in clonorchiasis [2,40,42], the association was not demonstrated in this study.

This study demonstrated the association between *C*. *sinensis* infection and enlargement of gallbladder. The individuals with *C*. *sinensis* infection showed higher length and width in gallbladder regardless of gender and age, compared to those without the infection. However, the difference was only significant in those aged over 45 years in female and over 30 years in male. The insignificance in low age and later occurrence in female could be attributable to the low infection intensity in these populations. The insignificance in male aged over 45 years is argued to be caused by the limited sample size in negative cases. The enlargement of gallbladder weakens its function, which could also promote the formation of cholelithiasis [10]. This is consistent to the finding on the association between *C*. *sinensis* infection and cholelithiasis mentioned above.

Severe *A*. *lumbricoides* infection could cause hepatic ascariasis and biliary ascariasis, in which diverse hepatobiliary damages are also caused [43]. However, the prevalence of ascariasis was very low in this study, which avoided the potential interference on assessment on the association between *C*. *sinensis* infection and morbidity.

This study had several limitations. Firstly, the low prevalence of some morbidity indicators and limited sample size might lead to false non-association between these morbidity indicators and *C*. *sinensis* infection. Additionally, the sample size of control (without clonorchiasis) in male older than 45 years was quite small, which probably caused the nonsignificant results in the gallbladder size between those with and without infections. Secondly, *C*. *sinensis* infection is associated with severe morbidity as demonstrated in this study and thus some individuals in control probably had been infected by *C*. *sinensis* and then treated. Although *C*. *sinensis* infection had been cured, the hepatobiliary damages might still persist in imaging [44]. In this case, the effect between *C*. *sinensis* infection and hepatobiliary morbidity is underestimated. Thirdly, the subjective symptoms have not been evaluated in this study. They are difficult to be measured and thus we only assessed the objective presentations and sonographic appearances. Fourthly, follow-up studies are expected in future, which could observe the long-term outcomes, especially the association with cholangiocarcinoma.

Overall, it is demonstrated that significant hepatobiliary morbidity is associated with *C*. *sinensis* infection in this study. There also exist increasing trends by infection intensity in some morbidity. Our study justifies the reasonability of morbidity control for clonorchiasis in current stage because of high infection and intensity as well as subsequent morbidity. Intervention measurements are needed in clonorchiasis endemic areas. The association between *C*. *sinensis* infection and diverse morbidity could be used to persuade those individuals eating raw freshwater fish to give up the practice and inform the policy-makers to implement interventions.

## Supporting information

S1 Table Association of diarrhoea and infection with *Clonorchis sinensis*. (DOCX)

S2 Table Morbidity demonstrating no association with *Clonorchis sinensis* infection in both univariable and multivariable logistic regression analysis. (DOCX)

S3 Table Association of fatty liver and infection with *Clonorchis sinensis*. (DOCX)

S4 Table Association of cholelithiasis and infection with *Clonorchis sinensis*. (DOCX)

S5 Table Association of gallbladder stone and infection with *Clonorchis sinensis*. (DOCX)

S6 Table Association of any bile duct dilatation and infection with *Clonorchis sinensis*. (DOCX)

S7 Table Association of intrahepatic bile duct dilatation and infection with *Clonorchis sinensis*. (DOCX)

S8 Table Association of periductal fibrosis and infection with *Clonorchis sinensis*. (DOCX)

S9 Table Association of changes in gallbladder wall and infection with *Clonorchis sinensis*. (DOCX)

S1 STROBE Checklist (PDF)
