# Supplementary material for: Severe hepatobiliary morbidity is associated with Clonorchis sinensis infection: The evidence from a cross-sectional community study
Source: PLoS Negl Trop Dis. 2021 Jan 28;15(1):e0009116. doi: 10.1371/journal.pntd.0009116 (PMC7880442; doi:10.1371/journal.pntd.0009116)
Supplement: S1 Table — (DOCX) [file pntd.0009116.s001.docx]

**S1 Table.** Association of diarrhoea and infection with *Clonorchis sinensis*

| **Factors** | | **No. participants** | **Diarrhoea** | | **Univariable regression** | | **Multivariable regression (1)^a^** | | **Multivariable regression (2)^b^** | |
| --- | --- | --- | --- | --- | --- | --- | --- | --- | --- | --- |
|  |  |  | **No.** | **Percentage (%)** | **cOR (95% CI)** | **P** | **aOR (95% CI)** | **P** | **aOR (95% CI)** | **P** |
| **Gender** | |  |  |  |  |  |  |  |  |  |
|  | **Female** | 370 | 28 | 7.6 | 1.0 |  | 1.0 |  | 1.0 |  |
|  | **Male** | 326 | 57 | 17.5 | 2.6 (1.6-4.2) | <0.001 | 1.7 (1.0-3.0) | 0.074 | 1.7 (0.9-3.2) | 0.082 |
| **Age groups (years)** | |  |  |  |  | 0.798 |  | 0.867 |  | 0.854 |
|  | **10-29** | 113 | 13 | 11.5 | 1.0 |  | 1.0 |  | 1.0 |  |
|  | **30-44** | 167 | 24 | 14.4 | 1.3 (0.6-2.7) | 0.488 | 1.1 (0.5-2.3) | 0.831 | 1.1 (0.5-2.3) | 0.831 |
|  | **45-59** | 224 | 25 | 11.2 | 1.0 (0.5-2.0) | 0.925 | 0.8 (0.4-1.8) | 0.654 | 0.8 (0.4-1.8) | 0.642 |
|  | **60+** | 192 | 23 | 12.0 | 1.0 (0.5-2.2) | 0.901 | 0.9 (0.4-1.9) | 0.788 | 0.9 (0.4-1.9) | 0.769 |
| **Alcohol drinking^c^** | |  |  |  |  |  |  |  |  |  |
|  | **No** | 364 | 29 | 8.0 | 1.0 |  | 1.0 |  | 1.0 |  |
|  | **Yes** | 330 | 56 | 17.0 | 2.4 (1.5-3.8) | <0.001 | 1.4 (0.8-2.5) | 0.207 | 1.5 (0.8-2.6) | 0.185 |
| ***C. sinensis* infection** | |  |  |  |  |  |  |  |  |  |
|  | **Negative** | 236 | 14 | 5.9 | 1.0 |  | 1.0 |  | - |  |
|  | **Positive** | 460 | 71 | 15.4 | 2.9 (1.6-5.3) | <0.001 | 2.0 (1.0-4.0) | 0.039 | - | - |
| ***C. sinensis* intensity** | |  |  |  |  | 0.003 |  | - |  | 0.174 |
|  | **Negative** | 236 | 14 | 5.9 | 1.0 |  | - |  | 1.0 |  |
|  | **Light** | 185 | 25 | 13.5 | 2.5 (1.2-4.9) | 0.009 | - |  | 2.2 (1.1-4.5) | 0.033 |
|  | **Moderate** | 158 | 24 | 15.2 | 2.8 (1.4-5.7) | 0.003 | - |  | 1.7 (0.8-3.9) | 0.184 |
|  | **Heavy** | 117 | 22 | 18.8 | 3.7 (1.8-7.5) | <0.001 | - |  | 2.2 (0.9-5.2) | 0.082 |
| **Total** | | 696 | 85 | 12.2 | - | - | - | - | - | - |

^a^ Gender, age groups, alcohol drinking and *C. sinensis* infection were all included in multivariable logistic regression model.

^b^ Gender, age groups, alcohol drinking and *C. sinensis* intensity were all included in multivariable logistic regression model.

^c^ Data were not provided in two persons.
