# Supplementary material for: Severe hepatobiliary morbidity is associated with Clonorchis sinensis infection: The evidence from a cross-sectional community study
Source: PLoS Negl Trop Dis. 2021 Jan 28;15(1):e0009116. doi: 10.1371/journal.pntd.0009116 (PMC7880442; doi:10.1371/journal.pntd.0009116)
Supplement: S2 Table — (DOCX) [file pntd.0009116.s002.docx]

**S2 Table.** Morbidity demonstrating no association with *Clonorchis sinensis* infection in both univariable and multivariable logistic regression analysis^a^

| **Morbidity** | | **No. uninfected** | **No. infected** | **No. participants** | **Percentage of infected (%)** | **cOR (95% CI)** | **P** |
| --- | --- | --- | --- | --- | --- | --- | --- |
| **Right upper quadrant pain** | **Neg** | 221 | 418 | 639 | 65.4 | 1.0 |  |
|  | **Pos** | 15 | 42 | 57 | 73.7 | 1.5 (0.8-2.7) | 0.209 |
| **Hepatomegaly** | **Neg** | 236 | 457 | 693 | 65.9 | 1.0 |  |
|  | **Pos** | 0 | 3 | 3 | 100.0 | NA^b^ | 0.995 |
| **Liver cyst** | **Neg** | 235 | 449 | 684 | 65.6 | 1.0 |  |
|  | **Pos** | 1 | 11 | 12 | 91.7 | 5.8 (0.7-44.9) | 0.095 |
| **Intrahepatic bile duct stone** | **Neg** | 235 | 453 | 688 | 65.8 | 1.0 |  |
|  | **Pos** | 1 | 7 | 8 | 87.5 | 3.6 (0.4-29.7) | 0.229 |
| **Extrahepatic bile duct dilatation** | **Neg** | 236 | 455 | 691 | 65.8 | 1.0 |  |
|  | **Pos** | 0 | 5 | 5 | 100.0 | NA^b^ | 0.995 |
| **Cholecystectomy** | **Neg** | 236 | 459 | 695 | 66.0 | 1.0 |  |
|  | **Pos** | 0 | 1 | 1 | 100.0 | NA^b^ | 0.995 |
| **Polyps of gallbladder** | **Neg** | 236 | 459 | 695 | 66.0 | 1.0 |  |
|  | **Pos** | 0 | 1 | 1 | 100.0 | NA^b^ | 0.995 |
| **Sludge of gallbladder** | **Neg** | 232 | 441 | 673 | 65.5 | 1.0 |  |
|  | **Pos** | 4 | 19 | 23 | 82.6 | 2.5 (0.8-7.4) | 0.100 |

^a^ Results of multivariable logistic regression analysis were not demonstrated.

^b^ NA: not available.
