# Supplementary material for: Severe hepatobiliary morbidity is associated with Clonorchis sinensis infection: The evidence from a cross-sectional community study
Source: PLoS Negl Trop Dis. 2021 Jan 28;15(1):e0009116. doi: 10.1371/journal.pntd.0009116 (PMC7880442; doi:10.1371/journal.pntd.0009116)
Supplement: S3 Table — (DOCX) [file pntd.0009116.s003.docx]

**S3 Table.** Association of fatty liver and infection with *Clonorchis sinensis*

| **Factors** | | **No. participants** | **Fatty liver** | | **Univariable regression** | | **Multivariable regression (1)^a^** | | **Multivariable regression (2)^b^** | |
| --- | --- | --- | --- | --- | --- | --- | --- | --- | --- | --- |
|  |  |  | **No.** | **Percentage (%)** | **cOR (95% CI)** | **P** | **aOR (95% CI)** | **P** | **aOR (95% CI)** | **P** |
| **Gender** | |  |  |  |  |  |  |  |  |  |
|  | **Female** | 370 | 41 | 11.1 | 1.0 |  | 1.0 |  | 1.0 |  |
|  | **Male** | 326 | 42 | 12.9 | 1.2 (0.8-1.9) | 0.464 | 0.9 (0.5-1.5) | 0.578 | 0.9 (0.5-1.6) | 0.696 |
| **Age groups (years)** | |  |  |  |  | 0.029 |  | 0.076 |  | 0.074 |
|  | **10-29** | 113 | 5 | 4.4 | 1.0 |  | 1.0 |  | 1.0 |  |
|  | **30-44** | 167 | 28 | 16.8 | 4.4 (1.6-11.6) | 0.003 | 3.5 (1.3-9.4) | 0.015 | 3.5 (1.3-9.5) | 0.015 |
|  | **45-59** | 224 | 28 | 12.5 | 3.1 (1.2-8.2) | 0.024 | 2.4 (0.9-6.5) | 0.086 | 2.4 (0.9-6.7) | 0.084 |
|  | **60+** | 192 | 22 | 11.5 | 2.8 (1.0-7.6) | 0.044 | 2.1 (0.8-5.8) | 0.157 | 2.1 (0.8-6.0) | 0.152 |
| **Alcohol drinking^c^** | |  |  |  |  |  |  |  |  |  |
|  | **No** | 364 | 38 | 10.4 | 1.0 |  | 1.0 |  | 1.0 |  |
|  | **Yes** | 330 | 45 | 13.6 | 1.4 (0.9-2.1) | 0.196 | 1.1 (0.6-1.8) | 0.858 | 1.1 (0.6-1.9) | 0.833 |
| ***C. sinensis* infection** | |  |  |  |  |  |  |  |  |  |
|  | **Negative** | 236 | 14 | 5.9 | 1.0 |  | 1.0 |  | - |  |
|  | **Positive** | 460 | 69 | 15.0 | 2.8 (1.5-5.1) | <0.001 | 2.7 (1.4-5.2) | 0.003 | - | - |
| ***C. sinensis* intensity** | |  |  |  |  | 0.009 |  | - |  | 0.032 |
|  | **Negative** | 236 | 14 | 5.9 | 1.0 |  | - |  | 1.0 |  |
|  | **Light** | 185 | 29 | 15.7 | 2.9 (1.5-5.8) | 0.002 | - |  | 2.8 (1.4-5.5) | 0.004 |
|  | **Moderate** | 158 | 23 | 14.6 | 2.7 (1.3-5.4) | 0.005 | - |  | 2.5 (1.1-5.7) | 0.023 |
|  | **Heavy** | 117 | 17 | 14.5 | 2.7 (1.3-5.7) | 0.009 | - |  | 2.5 (1.0-6.3) | 0.042 |
| **Total** | | 696 | 83 | 11.9 | - | - | - | - | - | - |

^a^ Gender, age groups, alcohol drinking and *C. sinensis* infection were all included in multivariable logistic regression model.

^b^ Gender, age groups, alcohol drinking and *C. sinensis* intensity were all included in multivariable logistic regression model.

^c^ Data were not provided in two persons.
