# Supplementary material for: Severe hepatobiliary morbidity is associated with Clonorchis sinensis infection: The evidence from a cross-sectional community study
Source: PLoS Negl Trop Dis. 2021 Jan 28;15(1):e0009116. doi: 10.1371/journal.pntd.0009116 (PMC7880442; doi:10.1371/journal.pntd.0009116)
Supplement: S4 Table — (DOCX) [file pntd.0009116.s004.docx]

**S4 Table.** Association of cholelithiasis and infection with *Clonorchis sinensis*

| **Factors** | | **No. participants** | **Cholelithiasis** | | **Univariable regression** | | **Multivariable regression (1)^a^** | | **Multivariable regression (2)^b^** | |
| --- | --- | --- | --- | --- | --- | --- | --- | --- | --- | --- |
|  |  |  | **No.** | **Percentage (%)** | **cOR (95% CI)** | **P** | **aOR (95% CI)** | **P** | **aOR (95% CI)** | **P** |
| **Gender** | |  |  |  |  |  |  |  |  |  |
|  | **Female** | 370 | 20 | 5.4 | 1.0 |  | 1.0 |  | 1.0 |  |
|  | **Male** | 326 | 33 | 10.1 | 2.0 (1.1-3.5) | 0.021 | 1.9 (0.9-3.8) | 0.071 | 1.8 (0.9-3.9) | 0.116 |
| **Age groups (years)** | |  |  |  |  | 0.173 |  | 0.337 |  | 0.358 |
|  | **10-29** | 113 | 4 | 3.5 | 1.0 |  | 1.0 |  | 1.0 |  |
|  | **30-44** | 167 | 10 | 6.0 | 1.7 (0.5-5.7) | 0.362 | 1.5 (0.5-5.2) | 0.488 | 1.5 (0.4-5.1) | 0.505 |
|  | **45-59** | 224 | 20 | 8.9 | 2.7 (0.9-8.0) | 0.080 | 2.3 (0.7-7.2) | 0.146 | 2.3 (0.7-7.3) | 0.157 |
|  | **60+** | 192 | 19 | 9.9 | 3.0 (1.0-9.0) | 0.052 | 2.5 (0.8-7.7) | 0.124 | 2.4 (0.8-7.7) | 0.134 |
| **Alcohol drinking^c^** | |  |  |  |  |  |  |  |  |  |
|  | **No** | 364 | 26 | 7.1 | 1.0 |  | 1.0 |  | 1.0 |  |
|  | **Yes** | 330 | 27 | 8.2 | 1.2 (0.7-2.0) | 0.607 | 0.7 (0.3-1.3) | 0.235 | 0.7 (0.3-1.3) | 0.214 |
| ***C. sinensis* infection** | |  |  |  |  |  |  |  |  |  |
|  | **Negative** | 236 | 8 | 3.4 | 1.0 |  | 1.0 |  | - |  |
|  | **Positive** | 460 | 45 | 9.8 | 3.1 (1.4-6.7) | 0.004 | 2.4 (1.0-5.7) | 0.044 | - | - |
| ***C. sinensis* intensity** | |  |  |  |  | 0.024 |  | - |  | 0.227 |
|  | **Negative** | 236 | 8 | 3.4 | 1.0 |  | - |  | 1.0 |  |
|  | **Light** | 185 | 15 | 8.1 | 2.5 (1.0-6.1) | 0.040 | - |  | 2.3 (0.9-5.6) | 0.079 |
|  | **Moderate** | 158 | 17 | 10.8 | 3.4 (1.4-8.2) | 0.005 | - |  | 2.8 (1.0-7.5) | 0.045 |
|  | **Heavy** | 117 | 13 | 11.1 | 3.6 (1.4-8.9) | 0.006 | - |  | 2.4 (0.8-7.2) | 0.113 |
| **Total** | | 696 | 53 | 7.6 | - | - | - | - | - | - |

^a^ Gender, age groups, alcohol drinking and *C. sinensis* infection were all included in multivariable logistic regression model.

^b^ Gender, age groups, alcohol drinking and *C. sinensis* intensity were all included in multivariable logistic regression model.

^c^ Data were not provided in two persons.
