# Supplementary material for: Severe hepatobiliary morbidity is associated with Clonorchis sinensis infection: The evidence from a cross-sectional community study
Source: PLoS Negl Trop Dis. 2021 Jan 28;15(1):e0009116. doi: 10.1371/journal.pntd.0009116 (PMC7880442; doi:10.1371/journal.pntd.0009116)
Supplement: S5 Table — (DOCX) [file pntd.0009116.s005.docx]

**S5 Table.** Association of gallbladder stone and infection with *Clonorchis sinensis*

| **Factors** | | **No. participants** | **Gallbladder stone** | | **Univariable regression** | | **Multivariable regression (1)^a^** | | **Multivariable regression (2)^b^** | |
| --- | --- | --- | --- | --- | --- | --- | --- | --- | --- | --- |
|  |  |  | **No.** | **Percentage (%)** | **cOR (95% CI)** | **P** | **aOR (95% CI)** | **P** | **aOR (95% CI)** | **P** |
| **Gender** | |  |  |  |  |  |  |  |  |  |
|  | **Female** | 370 | 18 | 4.9 | 1.0 |  | 1.0 |  | 1.0 |  |
|  | **Male** | 326 | 28 | 8.6 | 1.8 (1.0-3.4) | 0.051 | 2.0 (0.9-4.1) | 0.079 | 1.8 (0.8-4.1) | 0.145 |
| **Age groups (years)** | |  |  |  |  | 0.034 |  | 0.076 |  | 0.082 |
|  | **10-29** | 113 | 2 | 1.8 | 1.0 |  | 1.0 |  | 1.0 |  |
|  | **30-44** | 167 | 7 | 4.2 | 2.4 (0.5-11.9) | 0.274 | 2.2 (0.4-11.2) | 0.334 | 2.2 (0.4-10.9) | 0.354 |
|  | **45-59** | 224 | 18 | 8.0 | 4.9 (1.1-21.3) | 0.036 | 4.3 (1.0-19.5) | 0.058 | 4.2 (0.9-19.5) | 0.063 |
|  | **60+** | 192 | 19 | 9.9 | 6.1 (1.4-26.7) | 0.016 | 5.1 (1.1-23.2) | 0.034 | 5.0 (1.1-23.1) | 0.038 |
| **Alcohol drinking^c^** | |  |  |  |  |  |  |  |  |  |
|  | **No** | 364 | 24 | 6.6 | 1.0 |  | 1.0 |  | 1.0 |  |
|  | **Yes** | 330 | 22 | 6.7 | 1.0 (0.6-1.8) | 0.969 | 0.6 (0.3-1.2) | 0.159 | 0.6 (0.3-1.2) | 0.128 |
| ***C. sinensis* infection** | |  |  |  |  |  |  |  |  |  |
|  | **Negative** | 236 | 7 | 3.0 | 1.0 |  | 1.0 |  | - |  |
|  | **Positive** | 460 | 39 | 8.5 | 3.0 (1.3-6.9) | 0.008 | 2.2 (0.9-5.6) | 0.083 | - | - |
| ***C. sinensis* intensity** | |  |  |  |  | 0.031 |  | - |  | 0.235 |
|  | **Negative** | 236 | 7 | 3.0 | 1.0 |  | - |  | 1.0 |  |
|  | **Light** | 185 | 12 | 6.5 | 2.3 (0.9-5.9) | 0.092 | - |  | 1.9 (0.7-5.2) | 0.188 |
|  | **Moderate** | 158 | 16 | 10.1 | 3.7 (1.5-9.2) | 0.005 | - |  | 3.0 (1.1-8.6) | 0.040 |
|  | **Heavy** | 117 | 11 | 9.4 | 3.4 (1.3-9.0) | 0.014 | - |  | 2.2 (0.7-7.1) | 0.181 |
| **Total** | | 696 | 46 | 6.6 | - | - | - | - | - | - |

^a^ Gender, age groups, alcohol drinking and *C. sinensis* infection were all included in multivariable logistic regression model.

^b^ Gender, age groups, alcohol drinking and *C. sinensis* intensity were all included in multivariable logistic regression model.

^c^ Data were not provided in two persons.
