# Supplementary material for: Severe hepatobiliary morbidity is associated with Clonorchis sinensis infection: The evidence from a cross-sectional community study
Source: PLoS Negl Trop Dis. 2021 Jan 28;15(1):e0009116. doi: 10.1371/journal.pntd.0009116 (PMC7880442; doi:10.1371/journal.pntd.0009116)
Supplement: S6 Table — (DOCX) [file pntd.0009116.s006.docx]

**S6 Table.** Association of any bile duct dilatation and infection with *Clonorchis sinensis*

| **Factors** | | **No. participants** | **Any bile duct dilatation** | | **Univariable regression** | | **Multivariable regression (1)^a^** | | **Multivariable regression (2)^b^** | |
| --- | --- | --- | --- | --- | --- | --- | --- | --- | --- | --- |
|  |  |  | **No.** | **Percentage (%)** | **cOR (95% CI)** | **P** | **aOR (95% CI)** | **P** | **aOR (95% CI)** | **P** |
| **Gender** | |  |  |  |  |  |  |  |  |  |
|  | **Female** | 370 | 24 | 6.5 | 1.0 |  | 1.0 |  | 1.0 |  |
|  | **Male** | 326 | 85 | 26.1 | 5.1 (3.1-8.2) | <0.001 | 3.0 (1.7-5.3) | <0.001 | 2.0 (1.1-3.7) | 0.030 |
| **Age groups (years)** | |  |  |  |  | 0.093 |  | 0.141 |  | 0.360 |
|  | **10-29** | 113 | 10 | 8.8 | 1.0 |  | 1.0 |  | 1.0 |  |
|  | **30-44** | 167 | 30 | 18.0 | 2.3 (1.1-4.8) | 0.036 | 2.0 (0.9-4.5) | 0.088 | 1.7 (0.8-3.8) | 0.198 |
|  | **45-59** | 224 | 42 | 18.8 | 2.4 (1.1-4.9) | 0.020 | 2.4 (1.1-5.2) | 0.029 | 1.8 (0.8-3.9) | 0.165 |
|  | **60+** | 192 | 27 | 14.1 | 1.7 (0.8-3.6) | 0.182 | 1.6 (0.7-3.6) | 0.244 | 1.2 (0.5-2.8) | 0.628 |
| **Alcohol drinking^c^** | |  |  |  |  |  |  |  |  |  |
|  | **No** | 364 | 26 | 7.1 | 1.0 |  | 1.0 |  | 1.0 |  |
|  | **Yes** | 330 | 83 | 25.2 | 4.4 (2.7-7.0) | <0.001 | 2.1 (1.2-3.7) | 0.007 | 2.0 (1.1-3.5) | 0.016 |
| ***C. sinensis* infection** | |  |  |  |  |  |  |  |  |  |
|  | **Negative** | 236 | 13 | 5.5 | 1.0 |  | 1.0 |  | - |  |
|  | **Positive** | 460 | 96 | 20.9 | 4.5 (2.5-8.3) | <0.001 | 1.9 (0.9-3.7) | 0.072 | - | - |
| ***C. sinensis* intensity** | |  |  |  |  | <0.001 |  | - |  | <0.001 |
|  | **Negative** | 236 | 13 | 5.5 | 1.0 |  | - |  | 1.0 |  |
|  | **Light** | 185 | 16 | 8.6 | 1.6 (0.8-3.5) | 0.210 | - |  | 1.2 (0.5-2.7) | 0.638 |
|  | **Moderate** | 158 | 35 | 22.2 | 4.9 (2.5-9.6) | <0.001 | - |  | 2.2 (1.0-4.9) | 0.047 |
|  | **Heavy** | 117 | 45 | 38.5 | 10.7 (5.5-21.0) | <0.001 | - |  | 4.4 (1.9-10.1) | <0.001 |
| **Total** | | 696 | 109 | 15.7 | - | - | - | - | - | - |

^a^ Gender, age groups, alcohol drinking and *C. sinensis* infection were all included in multivariable logistic regression model.

^b^ Gender, age groups, alcohol drinking and *C. sinensis* intensity were all included in multivariable logistic regression model.

^c^ Data were not provided in two persons.
