# Supplementary material for: Severe hepatobiliary morbidity is associated with Clonorchis sinensis infection: The evidence from a cross-sectional community study
Source: PLoS Negl Trop Dis. 2021 Jan 28;15(1):e0009116. doi: 10.1371/journal.pntd.0009116 (PMC7880442; doi:10.1371/journal.pntd.0009116)
Supplement: S7 Table — (DOCX) [file pntd.0009116.s007.docx]

**S7 Table.** Association of intrahepatic bile duct dilatation and infection with *Clonorchis sinensis*

| **Factors** | | **No. participants** | **Intrahepatic bile duct dilatation** | | **Univariable regression** | | **Multivariable regression (1)^a^** | | **Multivariable regression (2)^b^** | |
| --- | --- | --- | --- | --- | --- | --- | --- | --- | --- | --- |
|  |  |  | **No.** | **Percentage (%)** | **cOR (95% CI)** | **P** | **aOR (95% CI)** | **P** | **aOR (95% CI)** | **P** |
| **Gender** | |  |  |  |  |  |  |  |  |  |
|  | **Female** | 370 | 24 | 6.5 | 1.0 |  | 1.0 |  | 1.0 |  |
|  | **Male** | 326 | 84 | 25.8 | 5.0 (3.1-8.1) | <0.001 | 3.0 (1.7-5.2) | <0.001 | 2.0 (1.1-3.7) | 0.031 |
| **Age groups (years)** | |  |  |  |  | 0.110 |  | 0.172 |  | 0.400 |
|  | **10-29** | 113 | 10 | 8.8 | 1.0 |  | 1.0 |  | 1.0 |  |
|  | **30-44** | 167 | 30 | 18.0 | 2.3 (1.1-4.8) | 0.036 | 2.0 (0.9-4.5) | 0.088 | 1.7 (0.8-3.9) | 0.194 |
|  | **45-59** | 224 | 41 | 18.3 | 2.3 (1.1-4.8) | 0.025 | 2.3 (1.1-5.0) | 0.036 | 1.7 (0.8-3.8) | 0.187 |
|  | **60+** | 192 | 27 | 14.1 | 1.7 (0.8-3.6) | 0.182 | 1.6 (0.7-3.6) | 0.245 | 1.2 (0.5-2.9) | 0.614 |
| **Alcohol drinking^c^** | |  |  |  |  |  |  |  |  |  |
|  | **No** | 364 | 26 | 7.1 | 1.0 |  | 1.0 |  | 1.0 |  |
|  | **Yes** | 330 | 82 | 24.8 | 4.3 (2.7-6.9) | <0.001 | 2.1 (1.2-3.6) | 0.008 | 2.0 (1.1-3.4) | 0.019 |
| ***C. sinensis* infection** | |  |  |  |  |  |  |  |  |  |
|  | **Negative** | 236 | 13 | 5.5 | 1.0 |  | 1.0 |  | - |  |
|  | **Positive** | 460 | 95 | 20.7 | 4.5 (2.4-8.2) | <0.001 | 1.9 (0.9-3.7) | 0.073 | - | - |
| ***C. sinensis* intensity** | |  |  |  |  | <0.001 |  | - |  | <0.001 |
|  | **Negative** | 236 | 13 | 5.5 | 1.0 |  | - |  | 1.0 |  |
|  | **Light** | 185 | 16 | 8.6 | 1.6 (0.8-3.5) | 0.210 | - |  | 1.2 (0.6-2.7) | 0.632 |
|  | **Moderate** | 158 | 35 | 22.2 | 4.9 (2.5-9.6) | <0.001 | - |  | 2.2 (1.0-4.9) | 0.044 |
|  | **Heavy** | 117 | 44 | 37.6 | 10.3 (5.3-20.3) | <0.001 | - |  | 4.3 (1.9-9.9) | <0.001 |
| **Total** | | 696 | 108 | 15.5 | - | - | - | - | - | - |

^a^ Gender, age groups, alcohol drinking and *C. sinensis* infection were all included in multivariable logistic regression model.

^b^ Gender, age groups, alcohol drinking and *C. sinensis* intensity were all included in multivariable logistic regression model.

^c^ Data were not provided in two persons.
