# Supplementary material for: Severe hepatobiliary morbidity is associated with Clonorchis sinensis infection: The evidence from a cross-sectional community study
Source: PLoS Negl Trop Dis. 2021 Jan 28;15(1):e0009116. doi: 10.1371/journal.pntd.0009116 (PMC7880442; doi:10.1371/journal.pntd.0009116)
Supplement: S8 Table — (DOCX) [file pntd.0009116.s008.docx]

**S8 Table.** Association of periductal fibrosis and infection with *Clonorchis sinensis*

| **Factors** | | **No. participants** | **Periductal fibrosis^a^** | | **Univariable regression** | | **Multivariable regression (1)^b^** | | **Multivariable regression (2)^c^** | |
| --- | --- | --- | --- | --- | --- | --- | --- | --- | --- | --- |
|  |  |  | **No.** | **Percentage (%)** | **cOR (95% CI)** | **P** | **aOR (95% CI)** | **P** | **aOR (95% CI)** | **P** |
| **Gender** | |  |  |  |  |  |  |  |  |  |
|  | **Female** | 368 | 107 | 29.1 | 1.0 |  | 1.0 |  | 1.0 |  |
|  | **Male** | 324 | 240 | 74.1 | 7.0 (5.0-9.7) | <0.001 | 5.3 (3.5-8.1) | <0.001 | 3.5 (2.3-5.5) | <0.001 |
| **Age groups (years)** | |  |  |  |  | <0.001 |  | 0.001 |  | 0.024 |
|  | **10-29** | 113 | 36 | 31.9 | 1.0 |  | 1.0 |  | 1.0 |  |
|  | **30-44** | 167 | 85 | 50.9 | 2.2 (1.3-3.7) | 0.002 | 2.4 (1.3-4.3) | 0.005 | 2.0 (1.1-3.6) | 0.025 |
|  | **45-59** | 221 | 115 | 52.0 | 2.3 (1.4-3.7) | <0.001 | 2.8 (1.6-5.0) | 0.001 | 2.1 (1.1-3.7) | 0.016 |
|  | **60+** | 191 | 111 | 58.1 | 3.0 (1.8-4.8) | <0.001 | 3.3 (1.8-6.0) | <0.001 | 2.5 (1.4-4.6) | 0.002 |
| **Alcohol drinking^d^** | |  |  |  |  |  |  |  |  |  |
|  | **No** | 362 | 129 | 35.6 | 1.0 |  | 1.0 |  | 1.0 |  |
|  | **Yes** | 328 | 217 | 66.2 | 3.5 (2.6-4.8) | <0.001 | 1.2 (0.8-1.8) | 0.334 | 1.1 (0.7-1.6) | 0.748 |
| ***C. sinensis* infection** | |  |  |  |  |  |  |  |  |  |
|  | **Negative** | 236 | 50 | 21.2 | 1.0 |  | 1.0 |  | - |  |
|  | **Positive** | 456 | 297 | 65.1 | 6.9 (4.8-10.0) | <0.001 | 3.2 (2.1-4.9) | <0.001 | - | - |
| ***C. sinensis* intensity** | |  |  |  |  | <0.001 |  | - |  | <0.001 |
|  | **Negative** | 236 | 50 | 21.2 | 1.0 |  | - |  | 1.0 |  |
|  | **Light** | 183 | 77 | 42.1 | 2.7 (1.8-4.1) | <0.001 | - |  | 2.1 (1.3-3.3) | 0.002 |
|  | **Moderate** | 158 | 116 | 73.4 | 10.3 (6.4-16.5) | <0.001 | - |  | 4.9 (2.9-8.4) | <0.001 |
|  | **Heavy** | 115 | 104 | 90.4 | 35.2 (17.5-70.5) | <0.001 | - |  | 13.3 (6.2-28.7) | <0.001 |
| **Total** | | 692 | 347 | 50.1 | - | - | - | - | - | - |

^a^ Data on periductal fibrosis were not provided in four persons.

^b^ Gender, age groups, alcohol drinking and *C. sinensis* infection were all included in multivariable logistic regression model.

^c^ Gender, age groups, alcohol drinking and *C. sinensis* intensity were all included in multivariable logistic regression model.

^d^ Data were not provided in two persons.
