# Supplementary material for: Severe hepatobiliary morbidity is associated with Clonorchis sinensis infection: The evidence from a cross-sectional community study
Source: PLoS Negl Trop Dis. 2021 Jan 28;15(1):e0009116. doi: 10.1371/journal.pntd.0009116 (PMC7880442; doi:10.1371/journal.pntd.0009116)
Supplement: S9 Table — (DOCX) [file pntd.0009116.s009.docx]

**S9 Table.** Association of changes in gallbladder wall and infection with *Clonorchis sinensis*

| **Factors** | | **No. participants** | **Changes in gallbladder wall** | | **Univariable regression** | | **Multivariable regression (1)^a^** | | **Multivariable regression (2)^b^** | |
| --- | --- | --- | --- | --- | --- | --- | --- | --- | --- | --- |
|  |  |  | **No.** | **Percentage (%)** | **cOR (95% CI)** | **P** | **aOR (95% CI)** | **P** | **aOR (95% CI)** | **P** |
| **Gender** | |  |  |  |  |  |  |  |  |  |
|  | **Female** | 370 | 42 | 11.4 | 1.0 |  | 1.0 |  | 1.0 |  |
|  | **Male** | 326 | 69 | 21.2 | 2.1 (1.4-3.2) | <0.001 | 2.4 (1.4-4.0) | 0.001 | 2.8 (1.6-5.0) | <0.001 |
| **Age groups (years)** | |  |  |  |  | 0.032 |  | 0.022 |  | 0.016 |
|  | **10-29** | 113 | 8 | 7.1 | 1.0 |  | 1.0 |  | 1.0 |  |
|  | **30-44** | 167 | 25 | 15.0 | 2.3 (1.0-5.3) | 0.049 | 2.5 (1.1-6.0) | 0.035 | 2.7 (1.1-6.6) | 0.024 |
|  | **45-59** | 224 | 40 | 17.9 | 2.9 (1.3-6.3) | 0.010 | 3.2 (1.4-7.4) | 0.006 | 3.5 (1.5-8.2) | 0.004 |
|  | **60+** | 192 | 38 | 19.8 | 3.2 (1.5-7.2) | 0.004 | 3.6 (1.6-8.3) | 0.003 | 3.9 (1.6-9.0) | 0.002 |
| **Alcohol drinking^c^** | |  |  |  |  |  |  |  |  |  |
|  | **No** | 364 | 51 | 14.0 | 1.0 |  | 1.0 |  | 1.0 |  |
|  | **Yes** | 330 | 60 | 18.2 | 1.4 (0.9-2.0) | 0.135 | 0.9 (0.5-1.4) | 0.587 | 0.9 (0.6-1.6) | 0.831 |
| ***C. sinensis* infection** | |  |  |  |  |  |  |  |  |  |
|  | **Negative** | 236 | 27 | 11.4 | 1.0 |  | 1.0 |  | - |  |
|  | **Positive** | 460 | 84 | 18.3 | 1.7 (1.1-2.8) | 0.021 | 1.0 (0.6-1.8) | 0.881 | - | - |
| ***C. sinensis* intensity** | |  |  |  |  | 0.024 |  | - |  | 0.104 |
|  | **Negative** | 236 | 27 | 11.4 | 1.0 |  | - |  | 1.0 |  |
|  | **Light** | 185 | 35 | 18.9 | 1.8 (1.0-3.1) | 0.033 | - |  | 1.3 (0.8-2.4) | 0.317 |
|  | **Moderate** | 158 | 22 | 13.9 | 1.3 (0.7-2.3) | 0.465 | - |  | 0.6 (0.3-1.2) | 0.164 |
|  | **Heavy** | 117 | 27 | 23.1 | 2.3 (1.3-4.2) | 0.005 | - |  | 0.9 (0.4-1.9) | 0.749 |
| **Total** | | 696 | 111 | 15.9 | - | - | - | - | - | - |

^a^ Gender, age groups, alcohol drinking and *C. sinensis* infection were all included in multivariable logistic regression model.

^b^ Gender, age groups, alcohol drinking and *C. sinensis* intensity were all included in multivariable logistic regression model.

^c^ Data were not provided in two persons.
